# Significance of tumour regression in lymph node metastases of gastric and gastro‐oesophageal junction adenocarcinomas

**DOI:** 10.1002/cjp2.169

**Published:** 2020-05-13

**Authors:** Daniel Reim, Alexander Novotny, Helmut Friess, Julia Slotta‐Huspenina, Wilko Weichert, Katja Ott, Bastian Dislich, Sylvie Lorenzen, Karen Becker, Rupert Langer

**Affiliations:** ^1^ Department of Surgery Klinikum Rechts der Isar, TUM School of Medicine Munich Germany; ^2^ Institute of Pathology Technische Universität München Munich Germany; ^3^ RoMed Klinikum Rosenheim Rosenheim Germany; ^4^ Institute of Pathology University of Bern Bern Switzerland; ^5^ 3rd Department of Internal Medicine, Hematology/Medical Oncology Klinikum rechts der Isar, TUM School of Medicine Munich Germany

**Keywords:** gastric cancer, lymph nodes, tumour regression, neoadjuvant chemotherapy

## Abstract

The presence of lymph node (LN) metastases is one of the most important negative prognostic factors in upper gastrointestinal carcinomas. Tumour regression similar to that in primary tumours can be observed in LN metastases after neoadjuvant therapy. We evaluated the prognostic impact of histological regression in LNs in 480 adenocarcinomas of the stomach and gastro‐oesophageal junction after neoadjuvant chemotherapy. Regressive changes in LNs (nodular and/or hyaline fibrosis, sheets of foamy histiocytes or acellular mucin) were assessed by histology. In total, regressive changes were observed in 128 of 480 patients. LNs were categorised according to the absence or presence of both residual tumour and regressive changes (LN−/+ and Reg−/+). 139 cases were LN−/Reg−, 28 cases without viable LN metastases revealed regressive changes (LN−/Reg+), 100 of 313 cases with LN metastases showed regressive changes (LN+/Reg+), and 213 of 313 metastatic LN had no signs of regression (LN+/Reg−). Overall, LN/Reg categorisation correlated with overall survival with the best prognosis for LN−/Reg− and the worst prognosis for LN+/Reg− (*p* < 0.001). LN−/Reg+ cases had a nearly significant better outcome than LN+/Reg+ (*p* = 0.054) and the latter had a significantly better prognosis than LN+/Reg− (*p* = 0.01). The LN/Reg categorisation was also an independent prognostic factor in multivariate analysis (HR = 1.23; 95% CI 1.1–1.38; *p* < 0.001). We conclude that the presence of regressive changes after neoadjuvant treatment in LNs and LN metastases of gastric and gastro‐oesophageal junction cancers is a relevant prognostic factor.

## Introduction

The presence of lymph node (LN) metastases is one of the most important negative prognostic factors for oesophageal and gastric cancer patients after surgical resection [1, 2, 3, 4]. This is true both for patients who are treated by primary resection and for those who are treated with multimodal treatment including neoadjuvant chemotherapy followed by surgery [5, 6, 7]. Regression of the primary tumour is associated with favourable outcome after neoadjuvant therapy followed by surgery in gastric cancer [8]. Histopathological assessment of tumour regression of gastrointestinal carcinomas is usually performed on the primary tumours and the most commonly used grading systems solely include the evaluation of regression at the site of the primary tumour [9, 10]. Regressive changes, however, can also be observed in LN metastases [11, 12, 13] and the currently reported sole information about the absence or presence of LN metastases along with the number of affected LNs by UICC/AJCC TNM staging [14] may thus not mirror the specific clinical situation if previous LN metastases have regressed under preoperative chemotherapy. It is therefore not clear if ‘node negative’ patients who initially had LN metastases have a different prognosis when these metastases completely regress compared to those who never had metastases. Studies on oesophageal carcinomas have already demonstrated a prognostic impact of tumour regression in LN metastases [12, 15, 16, 17]. Although generally advocated by the pathologists' community [18], and suggested in a recent expert opinion paper [19], the documentation of regressive changes in LNs and LN metastases in gastric cancers is still not routinely performed. Therefore, data on this issue are available to a much lesser degree. The only larger study identified in the literature failed to show convincing evidence for a prognostic value of tumour regression in LN metastases in an Asian gastric cancer patient cohort [11, 20]. Using a well‐characterised cohort of 480 patients with locally advanced gastric and gastro‐oesophageal junction adenocarcinomas treated with neoadjuvant chemotherapy followed by gastrectomy from a Western high‐volume surgical centre [7, 21], we therefore investigated the impact of regression in LN metastases.

## Material and methods

### Patients

The study included 480 histologically proven gastro‐oesophageal(Siewert type II/III) and gastric cancers, staged cT2‐cT4cN_any_ cM0 by endoscopy, endoscopic ultrasound, and computed tomography of the chest and abdomen and subsequentially treated in the Surgical Department of the TUM School of Medicine between 1991 and 2007 by neoadjuvant chemotherapy followed by surgery. Siewert type I gastro‐oesophageal cancers, and non‐resectable‐metastatic disease, were not included. Neoadjuvant treatment consisted of either two preoperative cycles of cisplatin or oxaliplatin/leucovorin/5‐FU(PLF/OLF, *n* = 302), PLF + paclitaxel(PLF‐T,*n* = 34), etoposide/adriamycin/cisplatin (EAP, *n* = 66 patients) or modified platinum based regimens (other, *n* = 78). All surgical procedures were performed according to the Japanese guidelines for gastric cancer treatment including standardised D2‐LN dissection. For gastro‐oesophageal junction cancers (Siewert type II and III) the surgical procedure was extended to the distal oesophagus, either accomplished by a transhiatal gastrectomy approach or, if the oral resection margin determined by intraoperative frozen section was positive, by an Ivor‐Lewis procedure (abdominothoracic oesophagectomy). Patients were followed up every 6–12 months in the comprehensive cancer centre CCCM^TUM^ over the next 5 years by endoscopy and CT scans according to the institutional protocol. Survival was computed from the day of surgery. Institutional Review Board‐approval for this study was obtained according to local guidelines.

### TNM staging and pathological parameters

TNM staging was performed according to the eighth edition of the UICC/AJCC TNM classification [14]. For additional anatomic subclassification, the Siewert classification [22] was used. Tumour differentiation (grading) and histological subtyping were performed according to the WHO classification [23]. Data regarding histopathological tumour regression of the primary tumour were taken from previous studies [7, 21], using the tumour regression grading (TRG) system according to Becker [24].

### Evaluation of tumour regression in LN metastases

**Figure 1:**
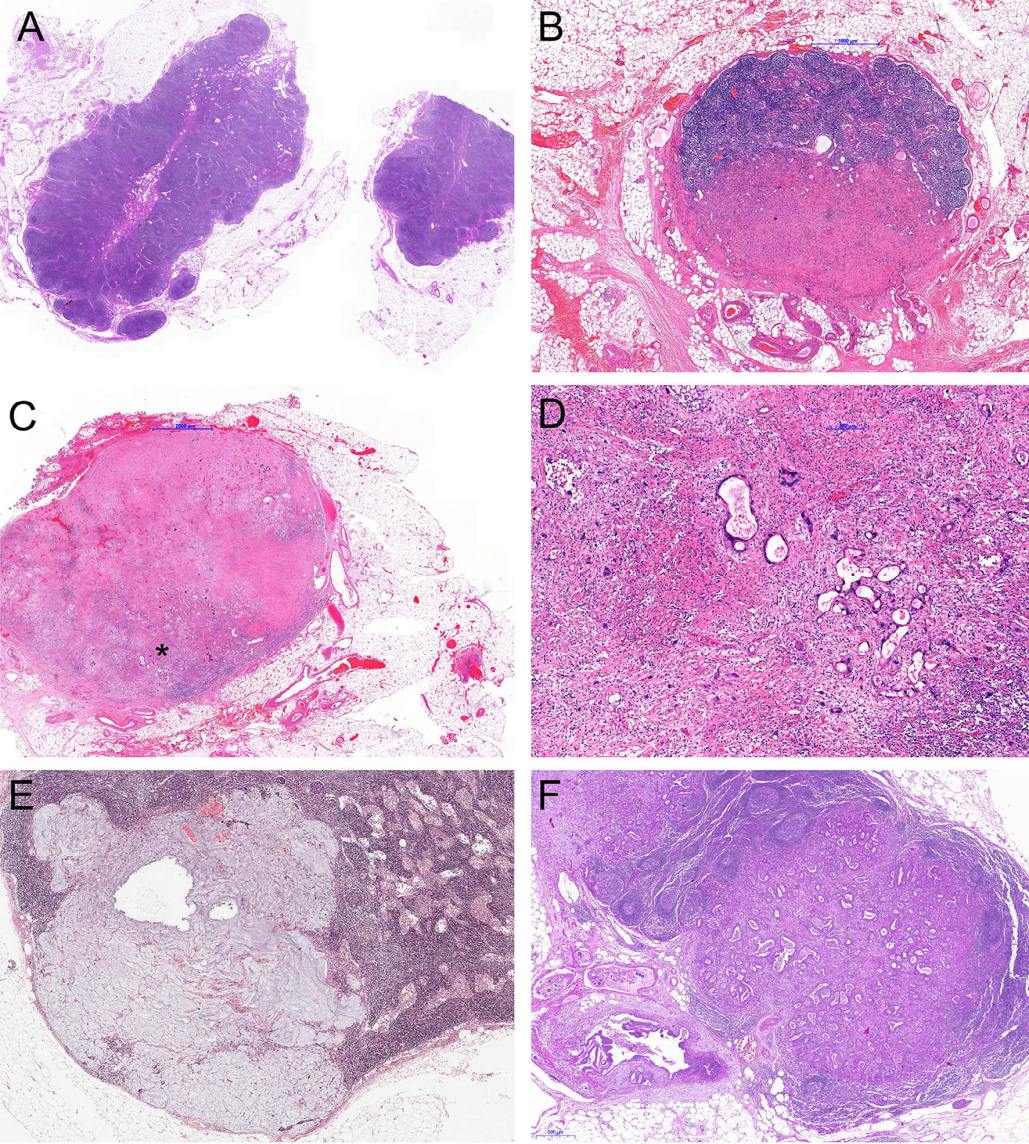
Examples of the LN/Reg categories: (A) LN−/Reg−, no regressive changes in a tumour free lymph node. (B) LN−/Reg+, regressive changes (hyaline fibrosis) without residual tumour. (C) LN+/Reg+, LN with regressive changes but with residual tumour (*marks area which is magnified in D). (D) Higher magnification of area marked * in (C). (E) acellular mucin in a lymph node as a sign of regression without residual tumour. (F) LN+/Reg−, Lymph node metastasis without regressive changes.

The histological work up of the resection specimens followed a standardised approach [24]. LNs were harvested and either completely embedded if the diameter was <0.5 cm or, otherwise, one section was taken of the largest diameter. In line with previous reports on oesophageal and gastric cancers, hyaline fibrosis, acellular mucin and the presence of sheets of foamy histiocytes were considered as characteristic signs of tumour regression [9, 10, 11, 20]. In cases where no visible tumour was detected but regressive changes were present, an additional three step sections were performed similar to the approach for the primary tumour [9]. Following the recent expert recommendation by Tsekrekos *et al* [19] the changes had to be detected in at least one LN, regardless of the number of affected nodes. The evaluation of the histological slides was performed prospectively by an experienced gastrointestinal pathologist (KB) at the time of the sign‐out of the surgical resection specimens. In line with other studies on gastric cancer and similar to the recommendation of Tsekrekos *et al* [19, 20], four categories of LNs were defined: negative LN without regressive signs (LN−/Reg−); negative LN with regressive signs (LN−/Reg+); metastatic LN with regressive signs (LN+/Reg+) and metastatic LN without regressive signs (LN+/Reg−) (Figure 1).

In order to investigate the interobserver agreement between pathologists, the LNs of an independent set of 30 cases with gastro‐oesophageal adenocarcinomas treated with neoadjuvant therapy at the Inselspital, University Hospital Bern, were assessed by two pathologists (BD, RL). The analyses included 60 slides with LNs with and without metastases, and the presence and absence of regressive changes in the LNs of each slide were scored by the two evaluators independently as described above.

### Statistical analysis

IBM SPSS Statistics 26 (IBM Corporation, Armonk, NY, USA) was used for statistical analysis. Comparisons between groups and categories were performed using crosstabs, χ^2^‐tests, and Fisher's exact tests. Survival analysis encompassed Kaplan–Meier curves andlog‐rank tests for univariate survival analysis and Cox regression analysis (enter method) for multivariate analysis. Interobserver agreement for the assessment of regressive changes was described using kappa values. *P* values of <0.05 were considered significant for all statistical tests.

## Results

### Patients

The study cohort of 480 patients was described in detail previously [7, 21]. The mean age of patients was 58 years (range: 17–78 years). One hundred and forty one patients were female and 339 male. The tumours were located in the gastro‐oesophageal junction (i.e. AEG Type II according to Siewert) in 177 cases. Three hundred and three cases were gastric cancer patients, with 83 patients having their tumours in the cardia/fundus region (i.e. AEG Type III according to Siewert) and 220 in the corpus and antrum, among them 21 patients with involvement of the whole stomach (see supplementary material, Table S1).

### Pathological data

Seventeen tumours (3.5%) had complete tumour regression (ypT0), 29 tumours (6%) were ypT1, 42 tumours (9%) ypT2, 239 tumours (50%) ypT3 and 153 tumours (32%) were in the ypT4 category. LN metastases were present in 313 cases (65%), the majority (148 cases) from the ypN1 category. Complete tumour resection was achieved in 374 cases (78%). Resectable distant metastases at the time of surgery were recorded in 72 cases (15%). Tumours were well to moderately differentiated in 88 cases (18%) and poorly differentiated in 392 cases (82%). According to the WHO classification, 230 tumours (48%) were tubular and/or papillary (including solid variants), 32 tumours (7%) were mucinous, 151 tumours (32%) poorly cohesive, 41 cases (95) mixed type and 26 cases (5%) other special types. Regression of the primary tumour was classified according to Becker as TRG1a (complete regression) in 17 cases (3.5%), TRG1b (<10% residual tumour) in 85 cases (18%), TRG2 (10–50% residual tumour) in 121 cases (25%) and TRG3 (>50% residual tumour) in 257 cases (53%) (see supplementary material, Table S1).

### Regressive changes in LNs

Interobserver agreement for the assessment of regressive changes in LN and LN metastases, respectively (absence versus presence) was excellent (kappa value 0.846) in the independent case series.

In the study cohort, LN count ranged from 4 to 147 (median = 30). Visible LN metastases were present in 313 of 480 cases (65%), ranging from 1 to 116 per case. Regressive changes were observed in a total of 128 of 480 patients (27%). Using the categorisation mentioned above, 139 patients had negative LNs without regression (LN−/Reg−; 29%). The remaining cases (331; 67%) had either completely regressed prior LN metastases (LN−/Reg+; *n* = 28; 6%), visible LN metastases with regressive changes (LN+/Reg+; *n* = 100; 21%) or LN metastases without regression (LN+/Reg−; *n* = 213; 44%).

### Correlation with pathological data

There was a significant correlation between the LN/Reg categorisation and the following factors (Table 1): ypT category (*p* < 0.001), ypN category (*p* < 0.001), distant metastases (*p* = 0.003), resection status (*p* < 0.001) and tumour differentiation (*p* < 0.001). There was also a highly significant correlation with TRG (*p* < 0.001), with for example 17 of 28 (60%) LN−/Reg+ cases in the TRG1b group (1–10% residual tumour) and 46 of 100 LN+/Reg+ cases (46%) in the TRG2 group (>10–50% residual tumour). However, some cases showed divergent response behaviour between primary tumour and LN metastases: 16 patients showed good response in the primary and no response in metastatic LNs; and 40 patients demonstrated regressive changes in LN metastases (two among them complete regression), but only little or no regression in the primary tumour. Interestingly, although there was no correlation between TRG of the primary tumour and the neoadjuvant chemotherapy regimen (PLF/OLF/PLF‐T versus EAP and others; *p* = 0.538), patients who were treated with PLD/OLF or PLF‐T more frequently demonstrated regressive changes in the LNs than patients treated with other chemotherapy regimens (*p* = 0.022).

**Table 1:** Lymph node status (LN)/regressive changes (Reg) and pathological parameters.

		LN status/regressive changes				Total	Significance
		LN−/Reg−	LN−/Reg+	LN+/Reg+	LN+/Reg−		
ypT category	ypT0	11	3	2	1	17	*p* < 0.001
	ypT1	16	6	3	4	29	
	ypT2	23	3	6	10	42	
	ypT3	63	15	57	104	239	
	ypT4	26	1	32	94	153	
ypN category	ypN0	139	28	0	0	167	*p* < 0.001
	ypN1	0	0	59	89	148	
	ypN2	0	0	23	77	100	
	ypN3	0	0	18	47	65	
Distant metastases	Absent	131	24	79	174	408	*p* = 0.003
	Present	8	4	21	39	72	
Resection status	R0	126	24	78	146	374	*p* < 0.001
	R1	13	4	22	67	106	
TRG (Becker)	1a	11	3	2	1	17	*p* < 0.001
	1b	39	17	14	15	85	
	2	37	6	46	32	121	
	3	52	2	38	165	257	
Differentiation (G)	1–2	38	9	18	23	88	*p* < 0.001
	3	11	19	82	190	392	
WHO subtype	Tub/pap/tubpap/sol	66	19	50	95	230	*p* = 0.050
	Mucinous	4	1	9	18	32	
	Poorly coh.	54	3	23	71	151	
	Mixed	9	3	13	16	41	
	Other	6	2	5	13	26	
Localisation	GE‐junction	52	14	44	67	177	*p* = 0.066
	Stomach	87	14	56	146	303	
CTX	PLF/OLF/PLF‐T	95	22	81	138	336	*p* = 0.022
	Other	44	6	19	75	144	
Total		139	28	100	213	480	

CTX, chemotherapy; G, grading; GE, gastro‐oesophageal; pap, papillary; poorly coh, poorly cohesive; sol, solid; TRG, tumour regression grade; tub, tubular; tubpap, tubulopapillary.

### Survival analysis

Survival data were available from 452 patients. Median follow‐up was 29 months (range 1–269 months), comprising 17 months (range 1–216 months) for survivors and 84 months (range 1–269) months for deceased patients. During the follow‐up period 288 patients (64%) died, the 5‐year survival rate was 40.1%, and the 10 year survival rate was 31.1%.

The following pathological parameters showed a prognostic impact in univariate analysis: ypT category, ypN category, presence of distant metastases, incomplete tumour resection, tumour grading, histological subtype according to Lauren and WHO with a negative impact of poorly cohesive morphology/diffuse type (*p* < 0.001 each). Tumour regression grade of the primary tumour also demonstrated a highly significant association with prognosis: TRG1 differed from TRG2 (*p* < 0.001; TRG1a versus TRG1b; *p* = 0.2) and TRG2 from TRG3 (*p* = 0.003), which showed even higher significant values in survival analysis compared to the original study with shorter follow‐up [21] (overall *p* < 0.001; HR = 1.757; 95% CI 1.514–2.041; see supplementary material, Figure S1).

**Figure 2:**
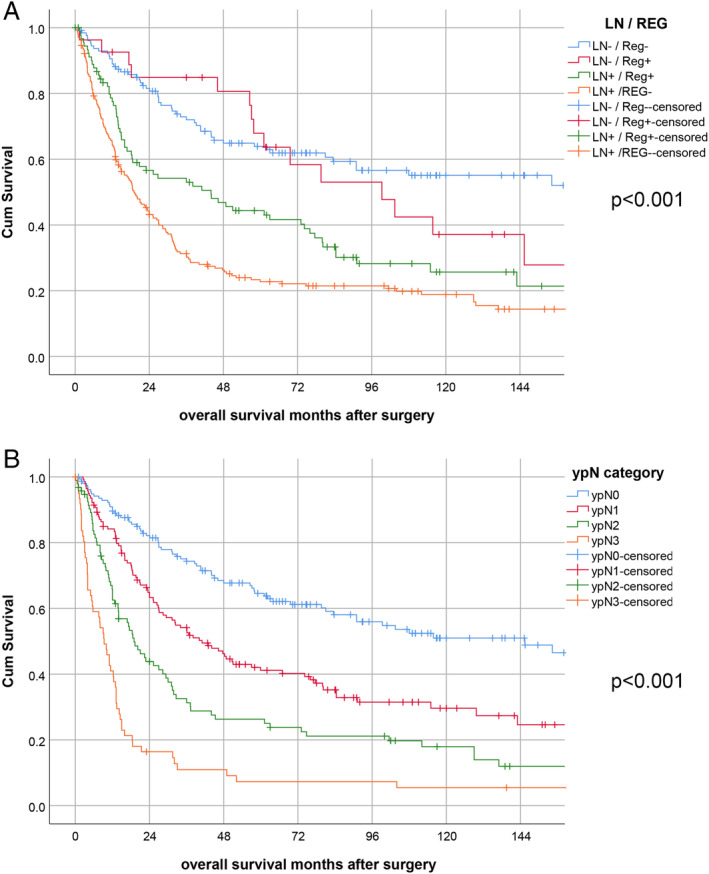
Survival analysis. (A) LN/Reg categories, (B) ypN category.

The LN/Reg‐categorisation was also prognostic regarding overall survival, with the best prognosis for LN−/Reg− and the worst prognosis for LN+/Reg− (overall *p* < 0.001; HR = 1.496; 95% CI 1.350–1.658). There was no significant difference between LN−/Reg− and LN−/Reg + cases (*p* = 0.3). However, LN−/Reg+ cases had a better outcome than LN+/Reg+ (*p* = 0.054) and the latter had a better prognosis than LN+/Reg− (*p* = 0.01; Figure 2).

The LN/Reg categorisation was also an independent prognostic factor in a multivariate model (HR = 1.23; 95% CI 1.1–1.38; *p* < 0.001; Table 2), encompassing ypT category, distant metastases, resection status, tumour differentiation and WHO subtype, respectively. Excluding patients with incomplete tumour resection and/or distant metastases from the survival analysis, leaving *n* = 338 patients (*n* = 316 for survival analysis) the results were almost identical (overall *p* < 0.001; HR = 1.534; 95% CI 1.353–1.738). Similar to the complete cohort there was no significant difference between LN−/Reg− and LN−/Reg+ cases (*p* = 0.206). LN−/Reg+ cases had a slightly but not significant better outcome than LN+/Reg+ (*p* = 0.133) but LN+/Reg− categories were associated with a better survival than LN+/Reg− (*p* = 0.049). In multivariate analysis, the LN/Reg categorisation was an independent prognostic factor (HR = 1.405; 95% CI 1.175–1.680; *p* < 0.001; see supplementary material, Figure S2 and Table S2) also for these patients.

**Table 2:** Multivariate analysis including LN status/regressive changes (LN/Reg).

	HR	95% CI for HR		*P* value
		Lower	Upper	
ypT category	1.310	1.093	1.570	0.003
LN/Reg category	1.230	1.099	1.378	<0.001
Tumour regression grade	1.266	1.055	1.520	0.011
Differentiation (grade)	1.037	0.792	1.359	0.791
WHO subtype	1.041	0.975	1.111	0.227
Distant metastases	1.361	1.008	1.838	0.044
Resection status	2.028	1.525	2.698	<0.001

The HR and the significance level of the survival analyses regarding the LN/Reg categorisation were, however, of a comparable value to the ypN category according to UICC/AJCC TNM classification, which showed very clear prognostic discrimination of four different groups (ypN0–ypN3; HR = 1.46; 95% CI 1.27–1.67; *p* < 0.001; Figure 2 and Table 3). Since these two systems show major overlaps between the categories, we did not perform a multivariate analysis including both LN/Reg and ypN categories.

**Table 3:** Multivariate analysis including ypN category.

	HR	95.0% CI for HR		Significance
		Lower	Upper	
ypT category	1.228	1.024	1.473	0.027
ypN category	1.456	1.272	1.666	<0.001
Tumour regression grade	1.259	1.052	1.506	0.012
Differentiation (grade)	0.976	0.742	1.283	0.859
WHO subtype	1.040	0.975	1.110	0.231
Distant metastases	1.298	0.960	1.754	0.090
Resection status	1.981	1.489	2.635	<0.0001

### Subgroup analysis: tumour location

**Figure 3:**
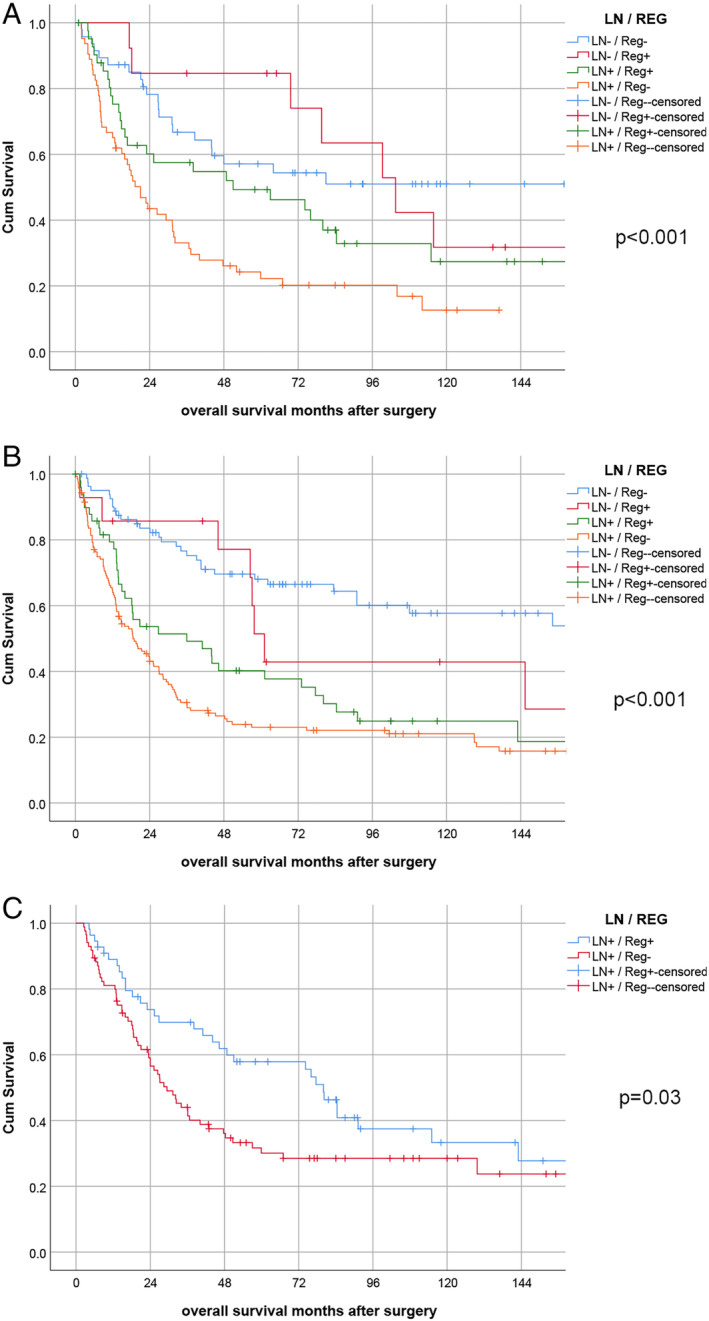
Survival analysis: subgroup analysis. (A) Subgroup analysis for LN/Reg categories in gastro‐oesophageal junction tumours. (B) Subgroup analysis for LN/Reg categories in gastric cancers. (C) Subgroup analysis for Reg categories for ypN1 patients.

Subgroup analysis according to the tumour location (gastro‐oesophageal junction/AEG II versus gastric cancer) revealed a similar prognostic impact of the LN/Reg categorisation for both tumour types (*p* < 0.001 each; Figure 3).

### Subgroup analysis: ypN categories

The most striking prognostic difference between the subcategories of the LN/Reg categorisation was observed in the ypN1 category (*p* = 0.030, Figure 3). For cases with ypN2 and higher there was no prognostic difference between the subgroups (*p* = 0.081).

## Discussion

We investigated the impact of regressive changes in LNs of gastric carcinomas and adenocarcinomas of the gastro‐oesophageal junction after neoadjuvant chemotherapy. Regressive changes in LNs were observed in around one quarter of the cases. A small proportion of patients demonstrated completely regressed former LN metastases, while mostly there were still visible metastatic infiltrates detectable despite regression. Most of the cases showed concordant response behaviour of the primary tumour and LN metastases. The presence of regressive changes in LN metastases was associated with a better survival compared to the absence of regression in LN metastases. Moreover, the patients with completely regressed prior LN metastases had an almost significant better outcome compared to patients with visible LN metastases with or without regression. A particular LN classification combining the presence or absence of tumour and regression showed independent prognostic value in a range comparable to the ypN category.

Our results contradict the findings of a previous study on gastric cancer by Zhu *et al* [20], who did not observe a significant impact of regressive changes in LN metastases although the authors used the same categorisation of regressive changes. Differences between this study and ours include a different ethnic background (China), a higher number of patients in our study and fewer cases with regressive changes in total although, in both studies, neoadjuvant platinum‐based chemotherapy was applied. Martin‐Romano*et al* [25] analysed tumour regression of the primary tumour and the LN metastases in a study that compared two different neoadjuvant therapy regimens. In line with our data they demonstrated that patients with complete regression in initially metastatic LNs had a better prognosis compared with those with residual metastases in their LNs. Similar to our study, there was no prognostic difference between patients with completely negative LNs and those with complete regression of a putatively former metastasis. The study of Martin‐Romano*et al*, however, lacks the comparison between positive LNs with and without regression, which showed the most striking difference in our analysis.

Data from oesophageal carcinomas that have been reported by several other groups also support the prognostic value of regressive changes in LNs [12, 15, 16, 17, 26]. These studies also indicate that the TNM ypN category may not sufficiently reflect the prognostic impact of LN metastases after neoadjuvant radiochemotherapy. Moreover, in line with our observations, intra‐observer agreement for the histopathological determination of regressive changes was very good [15]. Other entities in which the prognostic relevance of regression in LN metastases had a prognostic impact were breast [27] and rectal cancer [28].

We considered the presence of nodular hyaline fibrosis, sheets of foamy histiocytes and acellular mucin in LNs as signs of tumour regression. This has also been proposed by others [11, 20, 25], who could demonstrate that these findings are more frequently seen in treated tumours or metastases, compared to cases with primary resection. Our approach to determine the presence or absence of regression without further grading is in line with a recently proposed grading system for LN metastases of gastric cancers [19] and has been applied in a different study from China [20]. In addition, and in contrast to TRG in primary carcinomas, where it usually is possible to estimate the area of the previous tumour (the so‐called tumour bed) by macroscopy and histology, determination of the size of the initial LN metastases before treatment has to be considered less objectively. As we could also demonstrate excellent interobserver agreement for the determination of regressive changes, we think that this dichotomising approach is clear and reliable.

The limitations of this study are as follows. First, we did not specifically record the number of LNs with regressive changes. We could, however, demonstrate that the highest impact of regressive changes was detectable in the group of ypN1 categories which encompasses only 1–2 viable LN metastases. Moreover, regarding more detailed reporting on regression of LN metastasis, it should be also taken into account that elaborated reporting on each LN separately may also be impractical in daily routine practice. This has also been discussed in a recent survey about TRG in gastrointestinal cancers among gastrointestinal pathologists [18].

Second, this study also included adenocarcinomas of the gastro‐oesophageal junction/AEG type II which, according to the updated WHO classification, should now be grouped with oesophageal cancers. In our single centre cohort, however, the standardised treatment of these tumours was gastrectomy, and the chemotherapy regimen was the same for both locations [29], as opposed to current standards where adenocarcinomas of the gastro‐oesophageal junction/AEG type II are frequently treated by neoadjuvant chemoradiation instead of chemotherapy alone. In addition, the prognostic impact of the LN/Reg categorisation was similar for true gastric carcinomas and gastro‐oesophageal junction carcinomas, and our cohorts are, for both entities separately and in total, at least to our knowledge, the largest series investigating this topic so far. We therefore felt it appropriate to include both tumour types in an overall evaluation, while also presenting the data for both types in a separate analysis.

Third, a recent multicentre study showed the superiority of the FLOT protocol over other treatment regimens [30]. In this report, higher rates of favourable TRGs were reported in comparison to our single centre case collection where additional taxanes were administered in only a small subset of patients. In our study, however, regressive changes were observed more frequently in patients who were treated with PLF/OLF or PLF‐T than in patients treated with other platinum‐based chemotherapy regimens. This suggests that intensified preoperative treatment may also affect regression in LN metastases. In this context, it seems appropriate to prospectively collect data regarding regression in LNs and LN metastases in order to determine not only the prognostic impact but also the influence of modified treatment concepts.

In summary, our findings strongly support the prognostic impact of regressive changes in LN metastases of gastric and gastro‐oesophageal junction carcinomas. This warrants the recommendation that the presence of tumour regression in LN metastases should be routinely recorded in pathological reports of gastric and gastro‐oesophageal junction cancers.

## Author contributions statement

DR, AN, HF, JSH, WW, KO, BD, SL, KB and RL collected the data. RL carried out the statistical analysis. DR and RL drafted the manuscript.

## Supporting information


**Figure S1.** Impact of tumour regression grade in the primary tumour on survival
**Figure S2.** Impact of LN/Reg categorisation on survival in patients with completely resected tumours without distant metastases (R0M0)
**Table S1.** Patient cohort
**Table S2.** Multivariate analysis for R0M0 patients
