# Supplementary material for: Significance of tumour regression in lymph node metastases of gastric and gastro‐oesophageal junction adenocarcinomas
Source: J Pathol Clin Res. 2020 May 13;6(4):263–72. doi: 10.1002/cjp2.169 (PMC7578278; doi:10.1002/cjp2.169)
Supplement: Supplementary file 1 — Figure S1. Impact of tumour regression grade in the primary tumour on survival Figure S2. Impact of LN/Reg categorisation on survival in patients with completely resected tumours without distant metastases (R0M0) Table S1. Patient cohort Table S2. Multivariate analysis for R0M0 patients [file CJP2-6-263-s001.pdf]

Significance of tumour regression in lymph node metastases of gastric and gastro-oesophageal junction adenocarcinomas

D Reim *et al*, *J Pathol Clin Res*, DOI: 10.1002/cjp2.169

Supplementary Material

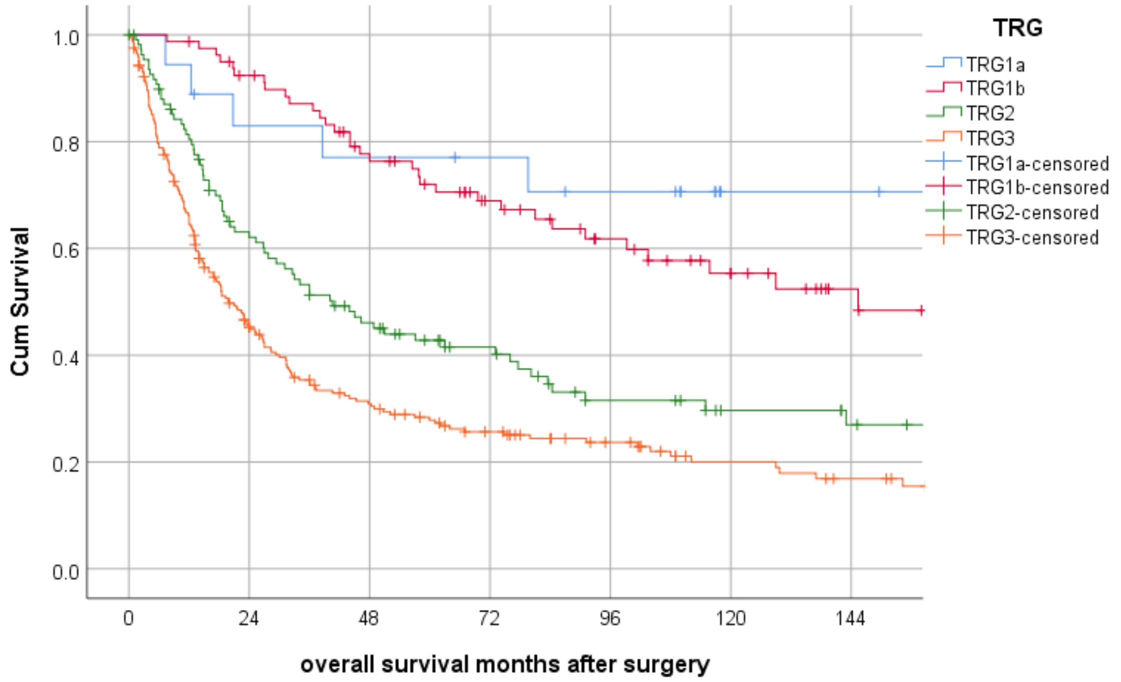

Figure S1. Impact of Tumour Regression Grade in the Primary Tumour on Survival ($p<0.001$)

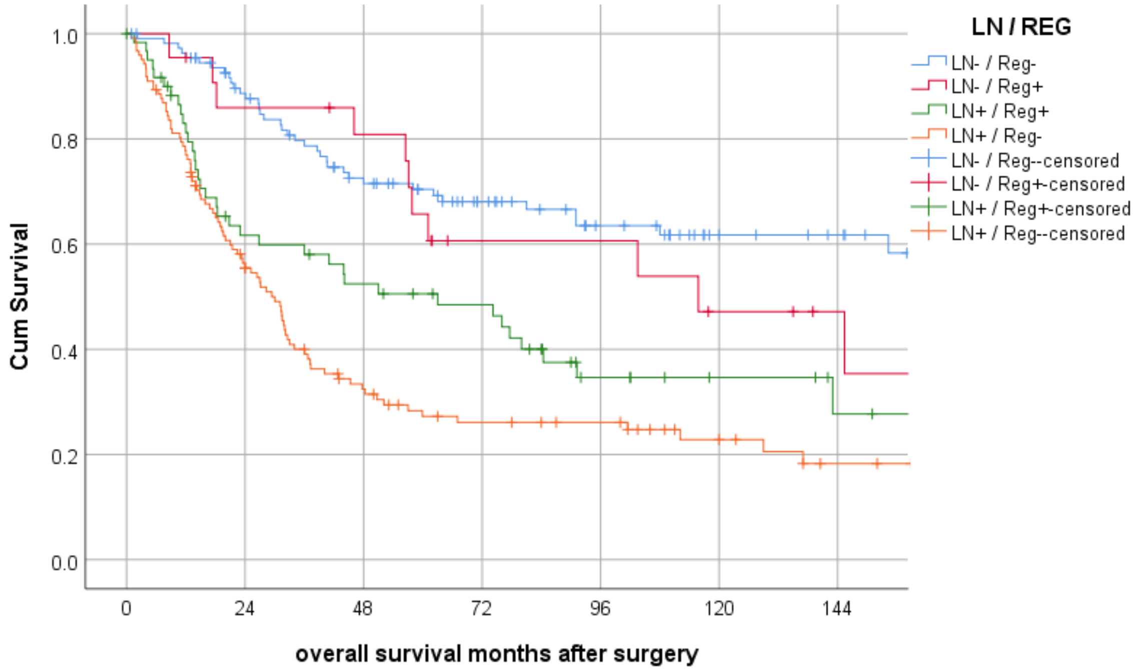

Figure S2. Impact of LN / Reg-Categorisation on Survival in Patients with Completely Resected Tumours without Distant Metastases (R0M0; $p<0.001$)

Table S1 Patient Cohort

		total	%
Gender	female	140	29
	male	340	71
Age	median 59 years (range; 17-78)		
ypT category	ypT0	17	4
	ypT1	29	5
	ypT2	42	9
	ypT3	239	50
	ypT4	153	32
ypN category	ypN0	167	45
	ypN1	148	31
	ypN2	100	21
	ypN3	65	13
Tumour Regression Grade (Becker)	TRG 1a	17	4
	TRG 1b	85	18
	TRG 2	121	24
	TRG 3	257	53
Distant metastases	absent	408	85
	present	72	15
Resection status	R0	374	78
	R1	106	22
Grading	1-2	88	18
	3	392	82
WHO Subtypes	tub/pap/tubpap/sol	230	48
	mucinous	32	7
	poorly cohesive	151	32
	mixed	41	9
	other	26	5
Localisation	GEJunction	177	37
	Stomach	303	63
Neoadjuvant CTX	PLF	302	63
	PLF-T	34	7
	EAP	66	14
	other	78	16
Total		480	100

Table S2. Multivariate Analysis for R0M0 Patients

	HR	95.0% CI for HR		p-value
		Lower	Upper	
ypTcategory	1.382	1.039	1.838	0.026
LN / Reg status	1.405	1.175	1.680	>0.001
Tumor Regression Grade	1.111	0.841	1.467	0.460
Differentiation (G)	1.151	0.760	1.742	0.507
WHO subtype	1.050	0.935	1.180	0.410
